# Surface hydrogenation regulated wrinkling and torque capability of hydrogenated graphene annulus under circular shearing

**DOI:** 10.1038/srep16556

**Published:** 2015-11-12

**Authors:** Yinfeng Li, Silin Liu, Dibakar Datta, Zhonghua Li

**Affiliations:** 1 Department of Engineering Mechanics, School of Naval Architecture, Ocean and Civil Engineering (State Key Laboratory of Ocean Engineering, Collaborative Innovation Center for Advanced Ship and Deep-Sea Exploration), Shanghai Jiao Tong University, Shanghai 200240, China; 2 School of Engineering, Brown University, Providence 02912, USA

## Abstract

Wrinkles as intrinsic topological feature have been expected to affect the electrical and mechanical properties of atomically thin graphene. Molecular dynamics simulations are adopted to investigate the wrinkling characteristics in hydrogenated graphene annulus under circular shearing at the inner edge. The amplitude of wrinkles induced by in-plane rotation around the inner edge is sensitive to hydrogenation, and increases quadratically with hydrogen coverage. The effect of hydrogenation on mechanical properties is investigated by calculating the torque capability of annular graphene with varying hydrogen coverage and inner radius. Hydrogenation-enhanced wrinkles cause the aggregation of carbon atoms towards the inner edge and contribute to the critical torque strength of annulus. Based on detailed stress distribution contours, a shear-to-tension conversion mechanism is proposed for the contribution of wrinkles on torque capacity. As a result, the graphane annulus anomalously has similar torque capacity to pristine graphene annulus. The competition between hydrogenation caused bond strength deterioration and wrinkling induced local stress state conversion leads to a U-shaped evolution of torque strength relative to the increase of hydrogen coverage from 0 to 100%. Such hydrogenation tailored topological and mechanical characteristics provides an innovative mean to develop novel graphene-based devices.

Possessing exceptional chemical^1^,^2^, thermal^3^, electrical^4^,^5^,^6^ and optical^7^ characteristics, graphene has attracted wide spread research attentions over the last few decades^8^,^9^,^10^. As a plate-like material consisting of a single layer of hexagonally arranged carbon atoms, graphene is easily susceptible to out-of-plane deformation and tends to form wrinkles and folds during fabrication and application^11^,^12^. These corrugations and wrinkles can be seen as a stability mechanism to relax the in-plane deformations of thin two dimensional materials^13^,^14^, and have been reported to affect the electronic and chemical properties of graphene by inducing effective magnetic fields and changing local potentials^15^,^16^,^17^. Wrinkled graphene is also reported to have increased chemical reactivity compared with flat regions, which can be utilized for tunable functionalization of graphene^18^. Thus, wrinkles in graphene have tremendous potential for design of flexible devices and stretchable electronics based on local strain^19^. For supported graphene under compressive strain, distributed wrinkles are affected by substrate adhesion and friction^20^. Chemically functionalized substrate with tunable adhesion has been utilized for the control of wrinkles in supported graphene^21^. For suspended graphene, surface chemical functionalization is an efficient method to introduce and control curvature^22^, folding^23^ and wrinkling^24^. Hydrogen adsorption on the surface of graphene, which is wildly used to modify properties including tunable band gap^25^, mechanical strength^26^ and thermal conductivity^27^,^28^, can cause membrane shrinkage and extensive membrane corrugation. It is necessary to understand the characteristics of wrinkling and folding in graphene tuned by hydrogenation for the design of graphene-based devices^19^.

Annular graphene as a popular structures in nanoelectronics^29^, is susceptible to wrinkling under circular shearing. In contrast to the wrinkling patterns that spread throughout rectangular graphene sheets, annular graphene shows unusual spiral wrinkling patterns surrounding the inner edge. Molecular dynamics and continuum mechanics have been applied to study the wrinkle characteristics and its effect on surface area of graphene^30^ The unusual wrinkles in annular graphene can be exploited for applications in nano-force sensors, tunable magnetic or electronic devices, as well as patterned stretchable electronics^31^ However, the applications of graphene always involve surface functionalization for specific optimized properties, while limited knowledge is available about the effect of surface characterization on wrinkling as well as torque capacity of annular graphene. In this work, we use MD simulations to investigate the wrinkling characteristics in hydrogenated graphene annulus under circular shearing at inner edge. The dependency of wrinkle amplitude and wrinkle numbers on hydrogenation are considered for an annulus with varying inner radius and hydrogen coverage. In particular, the effects of hydrogenation on the mechanical properties are considered by calculating the torque capability of annular graphene under two sets of boundary conditions, and the anomalous relationship between torque strength and hydrogenation enhanced wrinkling are reported.

## Results and Discussion

### Modeling of hydrogenated graphene annulus

**Figure 1:**
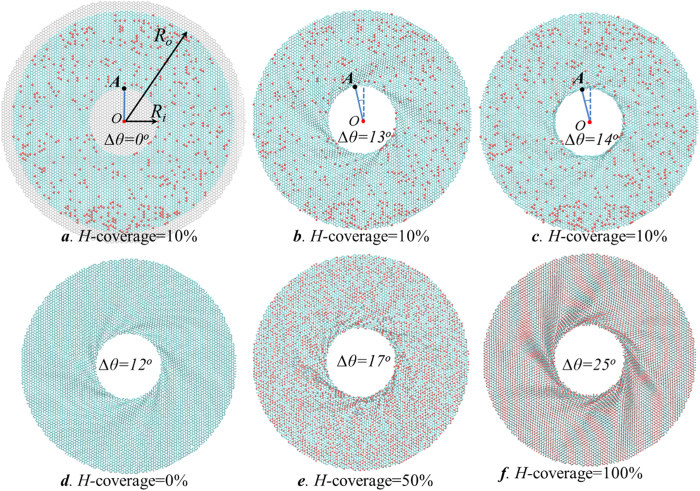
Annular graphene subjected to circular rotation at inner edge with inner radius *R*_i_ = 3 nm and outer radius *R*_o_ = 10 nm. (**a–c**) MD simulation snapshots of 10% hydrogenated annulus after different amount of rotation ∆*θ* at inner edge. The black point *A* at the inner edge serves as the reference point for rotation and the angle between the solid blue line and dash blue line illustrates the rotation angle ∆*θ*. (**a**) The gray domains represents the inner and outer boundaries which are not plotted in other figures for clarity. (**b**) The equilibrated wrinkled patterns around the inner rim at the onset of failure ∆*θ*_*c*_ = 13^o^. (**c)** The failure mode of the hydrogenated annulus at ∆*θ* = 14^o^. (**d–f**) The wrinkle configurations of annulus with different hydrogen coverage *H*-coverage=0, 50%, 100% at critical rotation angle ∆*θ*_*c*_.

The wrinkling pattern of pristine graphene annulus under circular shearing has been reported to focus in a neighborhood of the inner boundary and the individual wrinkles spiral around the center of the hole^31^. As shown in Fig. 1, we have constructed models of annular graphene with different hydrogen coverage in order to compare the wrinkling under uniform shearing around its edge. In order to compare the wrinkling of hydrogenated annular graphene under uniform shearing around its edge, annular graphene molecule dynamic models with different hydrogen coverage are constructed as depicted in Fig. 1. The atoms outside the radius *R*_*o*_ of the initial structure is defined as the outer boundary, and the atoms inside the inner radius *R*_*i*_ is the inner boundary (Fig. 1a). The annulus is composed of a circular graphene film with constrained boundaries as depicted by the gray domain in Fig. 1a. During relaxation process, boundary atoms are free to move in the plane of graphene annulus while the out-of-plane displacements are constrained by enforcing zero force and velocity along out-of-plane direction. The hydrogenated structure is relaxed by performing an energy minimization to the system, and the pre-stress of the constructed structure are relaxed iteratively by adjusting atom coordinates during energy minimization. The atoms between the inner and outer boundary *R*_*i*_ < *R* < *R*_*o*_ are free to move during the whole simulation. After relaxation, the atoms inside the inner boundary are rotated around the circle center as a rigid body at constant angular velocity 0.1 rad/*ps* until failure (Videos S1). The atoms at the outer boundary are fixed during rotation by enforcing zero force and velocity. The rotate angle of the inner boundary with respect to the fixed outer boundary is defined as the rotation angle of the graphene annulus ∆*θ* under circular shear (as illustrated in Fig. 1a–c). With the increase of rotation angle, the mechanical phenomena of pre-buckling, wrinkling, and failure of the annular graphene can be observed.

Figure 1a–c shows the snapshots from the dynamic rotation of the annulus with 10% hydrogen coverage. Hydrogen atoms are colored in red while the carbon atoms are colored in cyan. After inner edge rotate through an angle ∆*θ* (as illustrated in Fig. 1a–c), a series of spiral wrinkles concentrate around the inner rim of the annulus for the hydrogenated graphene annulus (Fig. 1b). The wrinkling of graphene annulus is qualified by wrinkle amplitude and wrinkle numbers (Refer to Figure S1 for illustration), where wrinkle amplitude represents the maximum wave amplitude of out-of plane deformation along *z*-direction, and the number of wrinkles concentrated around the inner edge is defined as wrinkle number. By comparing the equilibrated wrinkled patterns of annulus with different hydrogen coverage at critical rotation angle ∆*θ*_*c*_ (Fig. 1b,d–f), hydrogenated graphene annulus show different wrinkle characteristics with pristine graphene annulus. (Refer to Videos S1-S4 for the corresponding dynamic failure process) These tunable wrinkling characteristics provided by hydrogenation are sensitive to the changing of hydrogen coverage. Both the wrinkle amplitude and the critical rotation angle of annular graphene with higher hydrogen coverage are larger than these of annulus with lower hydrogen coverage.

### Hydrogenation regulated wrinkling and torque capability

**Figure 2:**
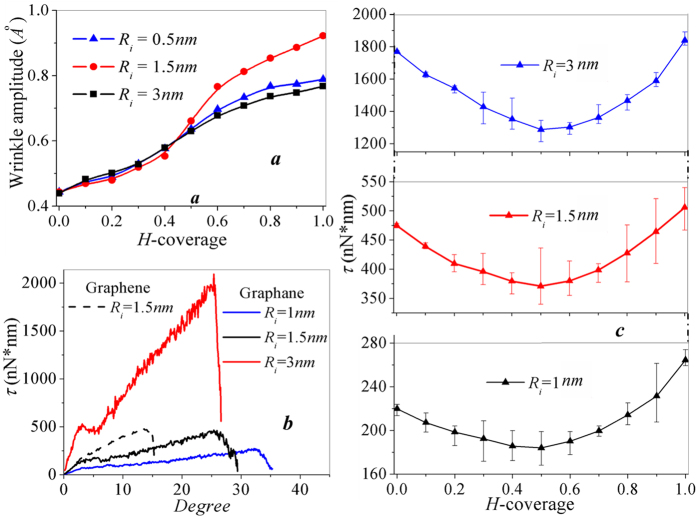
Effect of hydrogenation on wrinkle amplitude and torque capacity of graphene annulus under inner edge rotation. (**a**) The change of wrinkle amplitude with the increase of hydrogen coverage for three graphene annuluses with *R*_o_ = 10 *nm* and *R*_i_ = 1, 1.5, 3 *nm*; (**b**) Evolution of the torque in annulus as a function of rotate angle Δ*θ* during the whole rotation process; (**c**) the U-shaped change of torque strength versus the increase of hydrogen coverage for annulus with *R*_o_ = 10 *nm* and *R*_i_ = 1, 1.5, 3 *nm*.

Wrinkling characteristics of pristine annular graphene in terms of amplitude and number have been reported to dependent on inner radius *R*_i_ and rotation angle ∆*θ*^30^,^31^ The wrinkle configurations of graphene annulus at ∆*θ*_*c*_ with different hydrogen coverage shown in Fig. 1b,d–f also reveal the dependence of wrinkle characteristics on hydrogenation. Here, we systematically investigate the effect of hydrogenation on wrinkle characteristics and mechanical properties of annular graphene with different inner radius. In particular, we wish to know how wrinkle amplitude increases with hydrogen coverage. The wrinkling profile as a function of wave amplitude and wavelength depends on the magnitude of the circular shearing. Hence we record the evolution of amplitude during the rotation process and compare its maximum value for hydrogenated graphene with varying inner radius and hydrogen coverage. As shown in Fig. 2a, three group of annular graphene with inner radius *R*_i_ = 1 *nm*, 1.5 *nm*, 3 *nm* and *R*_o_ = 10 *nm* are considered when hydrogen coverage increase from 0 to 100%. Each point is the average value of maximum amplitude in five independent MD simulations. The maximum wrinkle amplitude increases with the hydrogen coverage, and the rate of increase depends on inner radius of annulus.

**Figure 3:**
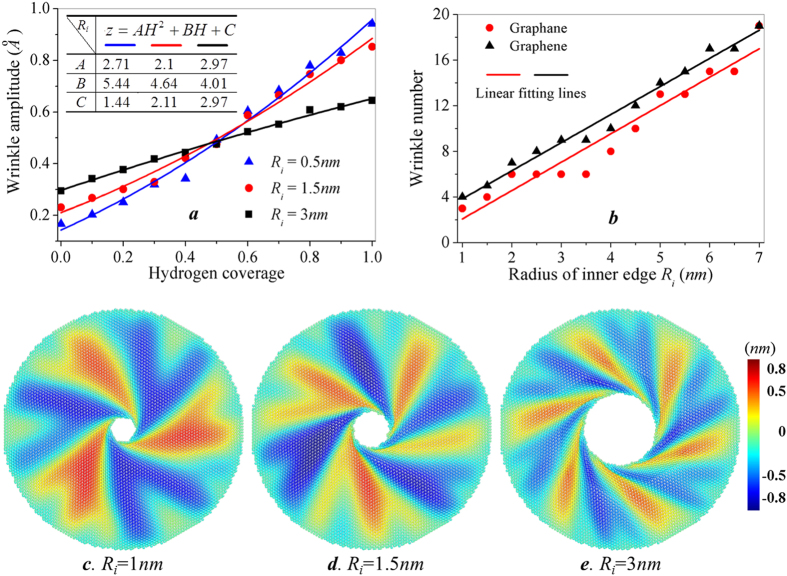
Effect of hydrogenation on the wrinkle characteristics of graphene annulus with different inner radius. (**a**) The amplitude of wrinkles as a function of hydrogen coverage for three graphene annuluses with *R*_o_ = 10 *nm* and *R*_i_ = 1, 1.5, 3 *nm*. (**b**) The number of wrinkles as a function of *R*_i_ for graphene and graphane annuluses at the onset of torsion failure. (**c*****–*****e**) Wrinkle amplitude contours of three graphane annuluses at critical rotation angle in MD simulations with *R*_o_ = 10 *nm* and *R*_i_ = 1, 1.5, 3 *nm*, respectively.

The constructed annular graphene models can also be used to determine its critical torque capacity by recording the torque *τ* in annulus as a function of the applied rotation angle ∆*θ*. The solid lines in Fig. 2b shows the recorded *τ*-∆*θ* curves of fully hydrogenated graphene annulus with inner radius *R*_i_ = 1 *nm*, 1.5 *nm* and 3 *nm*, respectively. The ultimate torque strength is defined at the point where the peak stress is reached. In order to verify the accuracy of our model, we recorded the *τ*-∆*θ* curve of pristine graphene annulus with *R*_i_ = 1.5 *nm*. As the dash line in Fig. 3a shows, the peak torque of the recorded *τ*-∆*θ* curve predicts a material strengths of 39 *N*/*m*, which agrees with the experimental value 42 ± 4 *N/m*^32^. The agreement confirms that our MD simulations are appropriate and reliable. By comparing the *τ*-∆*θ* curve of graphane annulus and graphene annulus with *R*_i_ = 1.5 *nm* (represented by the black solid line and dash line in Fig. 2b), fully hydrogenated graphene annulus exhibits a similar peak strength with that of pristine graphene annulus, and shows way improved flexibility (∆*θ* = 28°). It is contrary to our expectations that the torque strength of graphane annulus composed of weak *sp*^3^ bonds should be lower than that of graphene with strong *sp*^2^ bonds. In order to systematically reveal the effect of hydrogenation on torsion strength of graphene annulus, we further calculated the torque strength of graphene with given inner radius at varying hydrogen coverage from 0–100%. As shown in Fig. 2c, the evolution of torque strengths with hydrogen coverage follows a U-shaped trend. The strength first decreases with the increase of hydrogen coverage within the range of 0 < *H* < 50%, and reaches a minimum at *H* = 50%. When *H* > 50%, the torque strength anomalously increases with increasing hydrogen coverage. Each point of Fig. 3b–d is the average value of five independent simulations and the scale bar shows the deviation. Such U-shaped evolution of torque strength stands for all these considered cases *R*_i_ = 1, 1.5, 3 *nm*. We also consider the effect of the size of outer radius *R*_o_ on the torque strength of annulus with fixed inner radius *R*_i_, and the variation of torque strength is negligible for both graphene and graphane over a wide range of outer radius *R*_o_. (Refer to Figure S2 for details)

### Wrinkling characteristics of hydrogenated graphene annulus

After hydrogenation, the *sp*^2^ bond of pristine graphene will be converted to *sp*^3^ bonds, which have different bond length and strength. Under the adopted boundary condition, the radii of the inner and outer boundaries are allowed to change during relaxation. Thus, the surface area and details of the inner rim of the graphene annulus varies with the hydrogen coverage because of *sp*^3^ bond hybridization. In order to reveal the intrinsic effects of hydrogenation on wrinkle and the mechanism for the anomalous mechanical characteristics, we adopted another set of fixed boundary conditions for further simulation. For the fixed boundary conditions, the atoms of the outer and inner boundaries are constrained to keep still during the relaxation process and the atoms in between are free to move during relaxation. Such relaxation guarantees the boundary radius and edge details of annulus with different hydrogen coverage are all same as that of pristine graphene, especially for the inner radius where failure initiates. (Further detailed discussion on boundary conditions can be referred in Figure S3) After relaxation, the atoms inside the inner boundary are rotated around the circle center as a rigid body at constant angular velocity while the atoms of the outer boundary are remain fixed.

**Figure 4:**
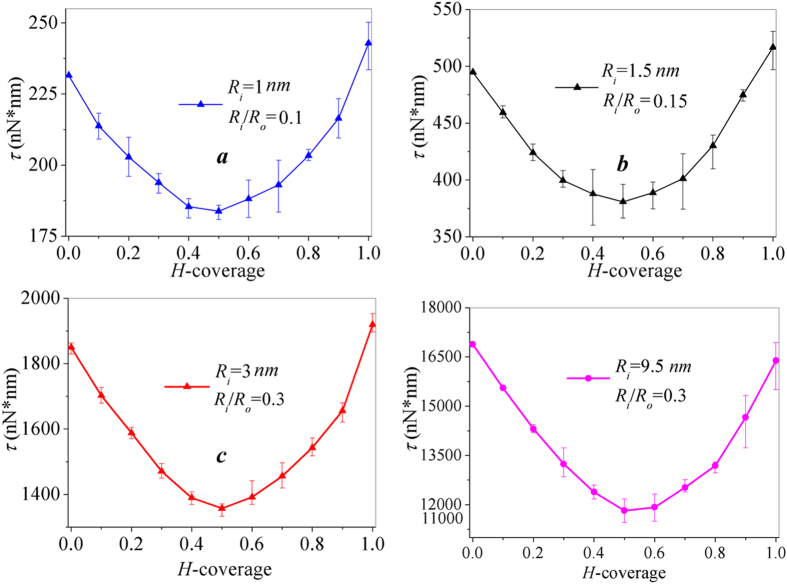
The U-shaped change of torque strength versus the increase of hydrogen coverage for annulus with different boundary radius *R*_o_ and *R*_i_. (**a**–**c**) *R*_o_ = 10 nm and *R*_i_ = 1, 1.5, 3 nm, respectively. (**d**) Larger scale graphene annulus with *R*_i_ = 9.5 nm and *R*_i_/*R*_o_ = 0.3.

We repeated the simulation shown in Fig. 2 under the fixed boundary conditions. (Refer to Videos S5 for the dynamics simulation process of graphene annulus with *H* = 0%, 10%, 50% and 100%, Figure S4 shows the corresponding snapshots of wrinkle configurations) Figs 3 and 4 show the evolution of wrinkle characteristics and torque capacity as a function of hydrogen coverage, and shows similarity to the results of the fully relaxed boundary conditions. Such similarity demonstrates the generality of the tunable effects of hydrogenation on graphene annulus. The fluctuation of results under fixed boundary condition is improved (Figure S5), and a more obvious trend can be found. As shown in Fig. 3, three groups of annular graphene are considered when hydrogen coverage increases from 0 to 100%. Each point in Fig. 3a is the average value of maximum amplitude in five independent MD simulations. The solid lines are the fitting curves of the results from simulation, and all follow a parabolic trend for these considered situations which can be expressed as:


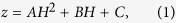


where *z* is the maximum wrinkle amplitude with given inner radius, *H* is the hydrogen coverage. *A*, *B* and *C* are the fitting coefficients to be determined from the calculated results of MD simulation, the value of these coefficients for the three groups of annulus are list in the table of Fig. 3a.

It is noticeable that the three curves depicted in Fig. 3a intersect as hydrogen coverage approximates to 50%. When *H-*coverage <50%, the graphene annulus with larger inner radius has larger wrinkle amplitude, which follows the same trend as that of pristine graphene reported in literature^30^. For annular graphene with hydrogen coverage larger than 50%, the wrinkle amplitude of graphene with small inner radius is anomalously larger than that of graphene with large inner radius. Fig. 3c–e shows the wrinkle configurations of hydrogenated graphane annulus (at hydrogen coverage *H* = 100%) before the onset of torsion failure. Each atom is colored according to the displacement in the out-of-plane direction, and the difference in color intensity reveals the difference in wrinkle amplitude. Annular graphane with *R*_i_ = 1 *nm* has the largest amplitude among these three situations while the graphane with *R*_i_ = 3 *nm* has the smallest wrinkle amplitude. Such anomalous trend of wrinkle amplitude tuned by hydrogen coverage provide innovative fabrication concept for flexible electronic devices and strain sensors.

Another important wrinkling characteristic is wrinkle number surrounding the inner edge. Here, we consider the wrinkle number of fully hydrogenated annular graphene with different inner radius. Figure 3b shows the comparison of wrinkle number between annular-graphene and -graphane presents at the onset of failure under circular rotation at inner edge. The solid lines are the fitting curves of simulated results. For a given inner radius, the wrinkle number of fully hydrogenated graphene is always less than that of pristine graphene. The wave number of pristine annular graphene increases almost linearly with the increasing inner radius, which is consistent with the reported investigations. It’s interesting to notice that the wave number of annular graphane also proportional to *R*_i_, and the linear fitting lines of graphene and graphane are parallel to each other. It can be concluded that hydrogenation enhanced flexibility increases the wrinkling amplitude and decreases the wrinkle number compared to the wrinkling characteristics of pristine graphene.

### Anomalous torque capacity tuned by hydrogenation

Figure 4a–c shows the torque strength of annulus with *R*_*o*_ = 10 *nm* and *R*_*i*_ = 1, 1.5, 3 *nm* at varying hydrogen coverage from 0–100% under fixed boundary conditions. The evolution of torque strengths versus hydrogen coverage still follows the U-shaped trend with a minimum value at *H* = 50%. We also consider a larger scale graphene annulus with *R*_i_ = 9.5 *nm* and *R*_i_/*R*_o_ = 0.3, which has the same *R*_i_/*R*_o_, and the surface area of the larger annulus is one order of magnitude larger than the model of Fig. 4c. The change of torque strength for larger annulus still follows a similar U-shaped trend. Thus, Fig. 4 demonstrates that the anomalous U-shaped torque capacity tuned by hydrogenation stands for graphene annulus of varying sizes.

**Figure 5:**
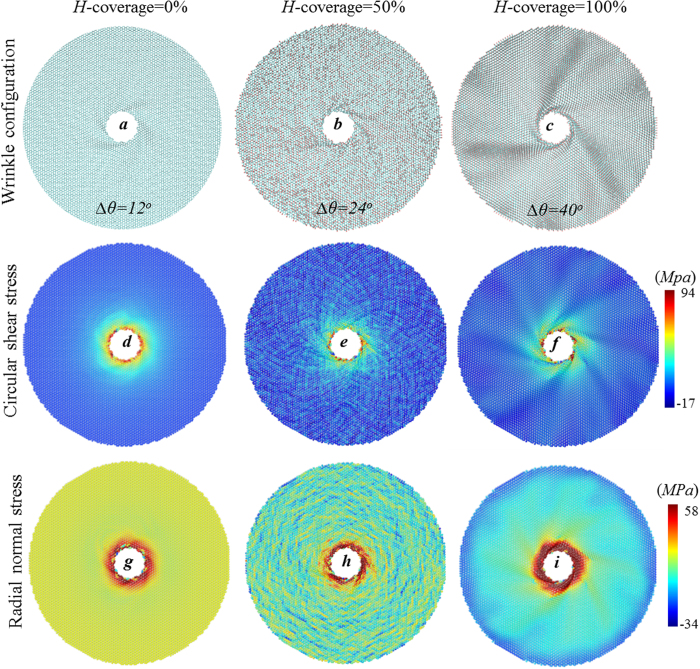
The wrinkling configurations for hydrogenated graphene annulus with *H*-coverage=0%, 50% and 100% at the critical torque angle Δ*θ*_*c*_ (**a**–**c**), and the corresponding stress distributions for circular shear stress (**d**–**f**) and radial normal stress (**g**–**i**).

It has been acknowledged that the critical tensile strength and fracture strain deteriorate with increasing hydrogen coverage is in the range of 0 < *H* < 100% because of the conversion of *sp*^2^ to *sp*^3^ bonding^33^,^34^. In order to explain the unexpected U-shaped evolution of torque strength versus hydrogen coverage, we further calculate the stress distributions during the dynamic rotating process. Figure 5 shows the wrinkle configurations at the onset of torsion failure for hydrogenated graphene annulus at *H* = 0%, 50% and 100%, as well as the corresponding distribution of radial normal stress and circular shear stress, respectively. The wrinkles are apparently enhanced by hydrogenation and the stress distribution is strongly affected by the wrinkle configurations, especially at the regions close to the inner edge where failure initiates. By comparing the stress contour of graphene annulus at H = 0% and 50%, the stress distribution of randomly hydrogenated graphene (Fig. 5e,h) are not as uniform as that of pristine graphene annulus(Fig. 5d,g) due to the hybridization of *sp*^3^ bond, stress of carbon atoms are lower than that of nearby hydrogenated carbon atoms. The torque capacity of annulus deteriorates dramatically as a result of the conversion of strong *sp*^2^ bonds to weak *sp*^3^ bonds as well as the nonuniform stress distribution.

By comparing the stress contour of annulus at *H* = 50% and 100%, the stress distribution of annulus with *H* = 100% is significantly affected by the enhanced wrinkle profiles. The circle shear stress field near the inner edge decays along the spiral wrinkle ridges. In addition, the ratio between the normal stress and the circular shear stress of graphene annulus changes with hydrogen coverage. The color intensity of these three shear stress contours (Fig. 5d–f) is approximately the same except for the distributions, while the color intensity of the normal stress contours (Fig. 5g–i) increases with the increase of hydrogen coverage. The increase in normal stress reveals the aggregation of carbon atoms towards inner edge because of the enhanced wrinkle amplitude (Refer to Videos S7 and S8 for more details). For pristine graphene annulus, the wrinkle amplitude is small and the sheets near the inner boundary are mainly under shear (Refer to Vides S6 for the dynamic failure). However, for hydrogenated graphene annulus with enhanced wrinkle amplitude, the sheets between wrinkle ridge and wrinkle valley are deformed vertically to the annulus plane, which mainly bear tension instead of shear. The stress distribution in Fig. 5f shows that the shear stress is high at the ridge and valley but small at the region in between. As the hydrogen coverage increase, the percentage of graphene bearing tension increases because of the elevated wrinkle amplitude. Since graphene has higher tensile strength than shear strength, such shear-to-tension conversion contributes to the torque capability of hydrogenated annulus. Thus, the torsion strength of hydrogenated graphene annulus is the result of competition between *sp*^3^ bond hybridization and wrinkle induced shear-to-tension local stress conversion. The wrinkle amplitude is relatively small when 0 < *H* < 50%, thus the effect of *sp*^3^ bond hybridization dominates the torsion capacity and the torsion strength decreases with the increase of hydrogen coverage. When 50% < *H* < 100%, the effect of wrinkle induced shear-to-tension stress distribution dominates and the torque capacity increases with *H* accordingly. The U-shaped evolution of torsion strength reveals the regulation of hydrogenation on the mechanical properties of graphene annulus, and illustrates the concept of creating graphene devices with designed mechanical and other properties by surface functionalization.

In conclusion, MD simulations have been performed to study the effects of hydrogenation on the wrinkle characteristics and torque capability of graphene annulus under circular shearing at inner edge. Two sets of boundary conditions are adopted to reveal the intrinsic characteristics and mechanism for the effect of hydrogenation. The wrinkle characters tuned by hydrogenation are first considered: wrinkle wave amplitude and numbers. The introduction of hydrogen converts the C-C bond from *sp*^2^ to *sp*^3^, and increase the flexibility of annulus. By changing the percentage of hydrogen atoms on the surface of graphene annulus with given inner radius, the amplitude increases parabolically with hydrogen coverage. The relationship between wrinkle amplitude and inner radius also show dependency on hydrogen coverage. The wrinkle number is found to increase with the radius of inner boundary linearly for both graphene annulus and graphane annulus, while the wrinkle number of graphane is smaller than that of graphene with same inner radius. The corresponding modifications in the mechanical properties are revealed by recording the torque in annulus versus rotate angle Δ*θ*. The torque strength decreases with the increase of hydrogen coverage when *H*-coverage is lower than 50%, then anomalously increase with hydrogen coverage when the coverage percent is higher than 50%. Annuluses with different inner radius and size scale all follow such U-shaped evolution of critical torque strength versus the hydrogen coverage. By calculating the distribution of the circular shear stress and radial normal stress, the stress distribution of partially hydrogenated graphene annulus is nonuniform, and the stress of pristine carbon atoms are noticed to be lower than that of nearby hydrogenated carbon atoms. Such nonuniform stress distribution and bond strength deterioration caused by introduction of hydrogen leads to the drop of the torque strength.

Another interesting phenomenon is the contribution of enhanced wrinkle amplitude to the torque strength of graphane. The radial normal stress contours demonstrate the carbon atoms of graphane annulus aggregate towards the inner edge due to the presents of enhanced wrinkles. As a result, the graphene domains between wrinkle ridge and valley will be tilted out of the annulus plane, and the tilted part bears tension instead of pure shear. Since graphene has higher tensile strength than shear strength, the elevated graphene amplitude will contribute to the torque capability. The competition between the hydrogenation caused bond strength deterioration and wrinkle induced local shear-to-tension conversion leads to the described U-shaped trend of torque strength with hydrogen coverage. Thus, our results demonstrate that the topological and mechanical characteristics of graphene annulus can be tailored with hydrogenation, the conclusions opens up a straightforward means to develop novel graphene -based devices.

## Methods

### Molecular dynamics simulation

We adopt the Adaptive Intermolecular Reactive Empirical Bond Order (AIREBO) force field^35^ in LAMMPS package^36^ to model the carbon-carbon and carbon-hydrogen interactions. Two sets of boundary conditions are adopted in the paper as described in main text, one has energy minimization for fully relaxation while the other constraint the boundaries during relaxation. Prior to rotating, the boundary atoms are relaxed for 2000 MD steps with time step *τ* *=* 0.1 *fs*, followed by another relaxation of 5000 MD steps with *τ* *=* 1 *fs*. After that, the atoms inside the inner boundary are rotated around the circle center as rigid body at constant angular velocity 0.1 rad/*ps* until failure (Videos S1-S4). The torque *M* imposed on the rigid rotating body, which equals to the torque in the domain *R*_*i*_<*R*<*R*_*o*_, is recorded during the rotating process at given constant angular velocity. The evolution of torque as a function of rotate angle is obtained to evaluate the torque capacity of annular graphene.

For hydrogenations in the domain between two concentric circles *R*_*i*_<*R*<*R*_*o*_, we first generated models of fully hydrogen functionalized annular graphene (*H-*coverage=100%) by bonding hydrogen atoms on both side of the graphene sheet alternatively (Fig. 1f). Further hydrogenated annular graphene sheets with certain hydrogen coverage were achieved by randomly removing hydrogen atoms from fully hydrogenated models as illustrated in Fig. 1a,d,e. Hydrogen atoms are colored in red while the carbon atoms are colored in cyan. All sets of the simulation were performed at room temperature under NVT ensemble.

## Additional Information

**How to cite this article**: Li, Y. *et al.* Surface hydrogenation regulated wrinkling and torque capability of hydrogenated graphene annulus under circular shearing. *Sci. Rep.* **5**, 16556; doi: 10.1038/srep16556 (2015).

## Supplementary Material

Supplementary Information

Supplementary Video S1

Supplementary Video S2

Supplementary Video S3

Supplementary Video S4

Supplementary Video S5

Supplementary Video S6

Supplementary Video S7

Supplementary Video S8
