# Supplementary material for: Surface hydrogenation regulated wrinkling and torque capability of hydrogenated graphene annulus under circular shearing
Source: Sci Rep. 2015 Nov 12;5:16556. doi: 10.1038/srep16556 (PMC4642312; doi:10.1038/srep16556)
Supplement: Supplementary Information [file srep16556-s1.doc]

**SUPPLEMENTARY INFORMATION**

[Surface hydrogenation](https://www.researchgate.net/publication/276173188_Effects_of_surface_atomistic_modification_on_mechanical_properties_of_gold_nanowires?ev=prf_pub) regulated wrinkling and torque capability of hydrogenated graphene annulus under circular shearing

Yinfeng Li^1,*^, Silin Liu^1^, Dibakar Datta^2^, Zhonghua Li^1^

^1^ Department of Engineering Mechanics, School of Naval Architecture, Ocean and Civil Engineering (State Key Laboratory of Ocean Engineering, Collaborative Innovation Center for Advanced Ship and Deep-Sea Exploration), Shanghai Jiao Tong University, Shanghai 200240, China

^2^ School of Engineering, Brown University, Providence 02912, USA

**The Electronic Supporting Information (ESI) includes seven videos:**

**Video S1**: The dynamic simulation process (including preliminary relaxation by energy minimization, further relaxation, rotation process) of hydrogenated graphene annulus under circular rotating at inner edge with hydrogen coverage *H*=10% and inner radius *R*_i_=3*nm*, *R*_o_=10*nm*.

**Video S2**: The dynamic simulation process of pristine graphene annulus under circular rotating at inner edge with inner radius *R*_i_=3*nm* and *R*_o_=10*nm*.

**Video S3**: The dynamic simulation process of hydrogenated graphene annulus under circular rotating at inner edge with hydrogen coverage *H*=50% and inner radius *R*_i_=3*nm*, *R*_o_=10*nm*.

**Video S4**: The dynamic simulation process of hydrogenated graphene annulus under circular rotating at inner edge with hydrogen coverage *H*=100% and inner radius *R*_i_=3*nm*, *R*_o_=10*nm*.

**Video S5**: The dynamic simulation process of hydrogenated graphene annulus under circular rotating at inner edge with hydrogen coverage *H*=100%, 50%, 10% and 0% and inner radius *R*_i_=3*nm*, *R*_o_=10*nm* under fixed boundary conditions.

**Video S6:** The wrinkling configurations of pristine graphene annulus under ircular rotating at inner edge with *R*_i_=3*nm*. The failure appears around the inner edge at a small rotation angle *∆θ*=10^o^. The wrinkle amplitude is small and no apparent aggregation occurs during the rotation process.

**Video S7**: Dynamic aggregation of carbon atom towards the inner edge of hydrogenated annulus with hydrogen coverage *H*=50% and *R*_i_=3*nm*.

**Video S8**: Dynamic aggregation of carbon atom towards the inner edge of hydrogenated annulus with hydrogen coverage *H*=100% and *R*_i_=3*nm*.


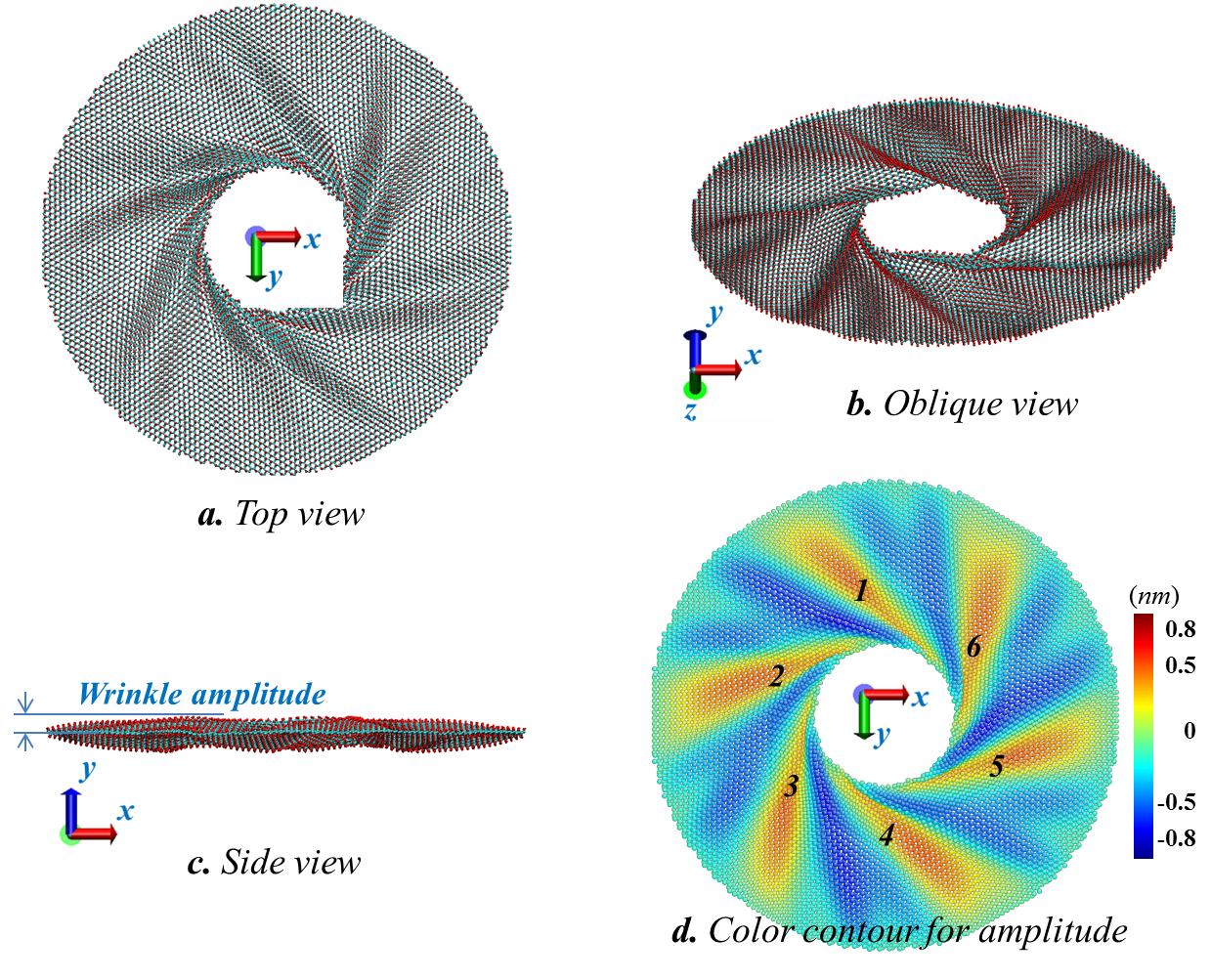


**Figure S1 | Illustration for the wrinkle amplitude and wrinkle number.** (***a*-*c***) The wrinkling configurations for hydrogenated graphene annulus with H-coverage=100% at the critical rotation angle of bond breaking Δ*θ_c_*=30*^o^* from different perspectives (***a.*** Top view, ***b.*** Oblique view, ***c.*** Side view). (***d***) The corresponding color contour for the wrinkle amplitude by coloring each atom according to the displacement in the *z*-direction, and the wrinkles are numbered as shown in the contour.


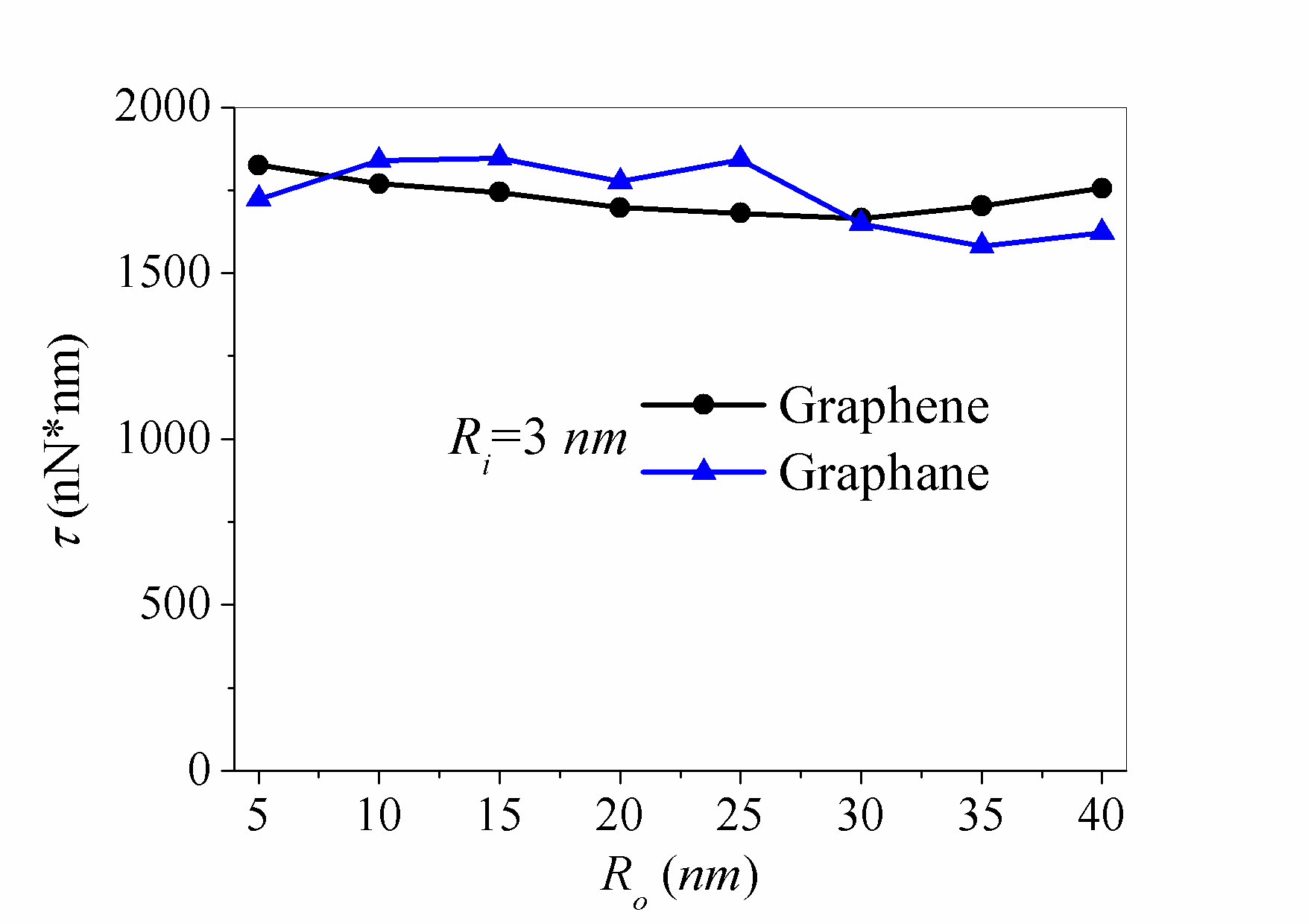


**Figure S2 | The change of torque strength versus the outer radius of graphene annulus *R*_o_ with fixed inner radius *R*_i_ =3*nm* for graphene and graphane.** The variation of torque strength is relatively negligible for both graphene and graphane, thus we only consider the annulus with outer radius *R*_0_ =10*nm* in the main text for the sake of calculate efficiency and simulation runtime.


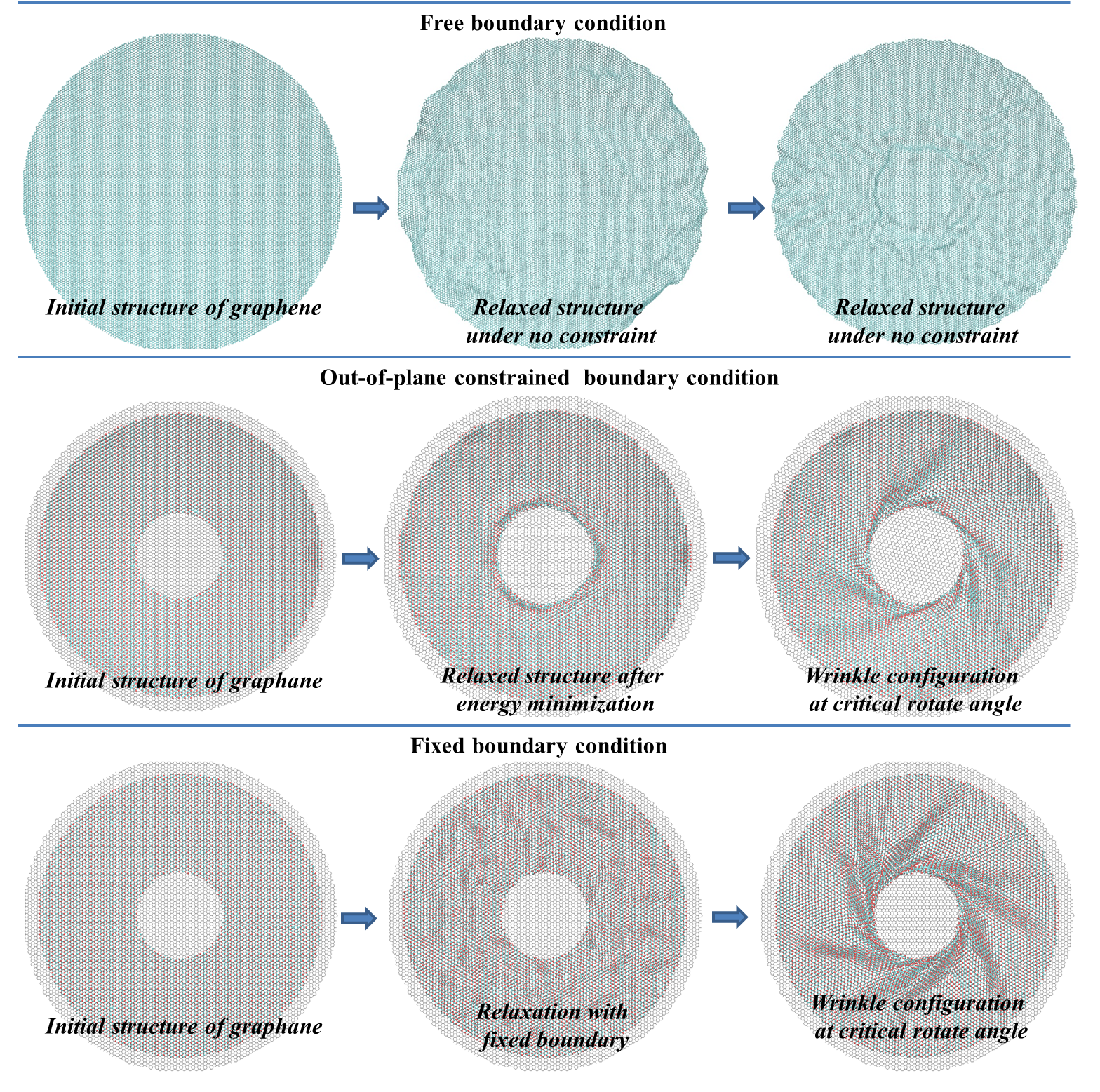


**Figure S3 | Illustrations for the two sets of boundary conditions adopted in the main text.** As the first row shows, the edge will be curved or folded during relaxation under totally free boundary condition which is not suitable for further rotation process. Thus the boundary should be constrained to deform in the plane of annulus during relaxation. For the second row, the edge are free to move in the plane during relaxation under energy minimization, and the structure are well relaxed with no pre-stress or strain. For the fixed boundary as shown in the third row, the inner and outer boundary are fixed to keep still during relaxation and the radius of the structure with different hydrogen coverage all have the same outer and inner radius during the whole simulation.


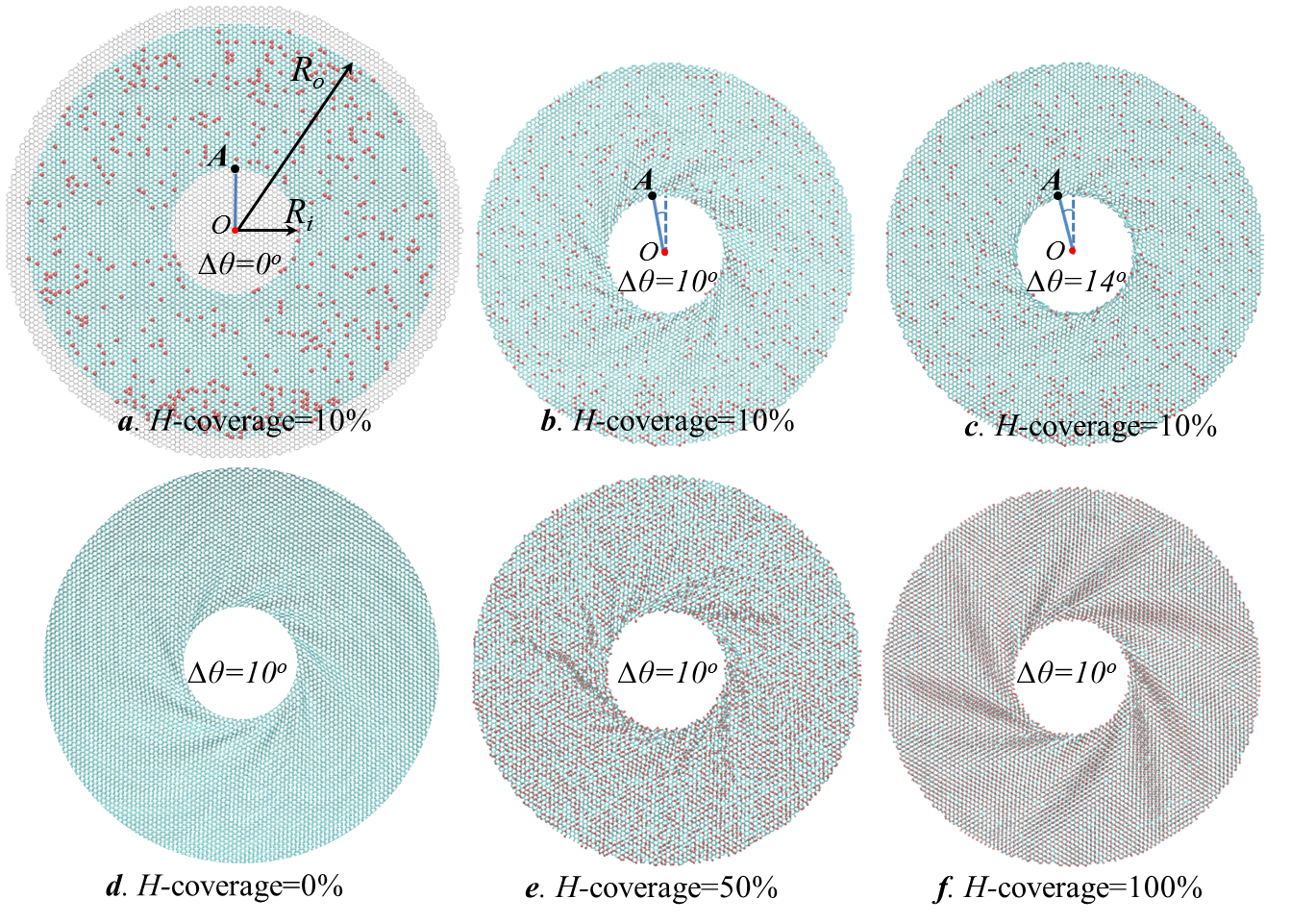


**Figure S4 | Annular graphene subjected to circular rotation at inner edge with inner radius *R*_i_=3nm and outer radius *R*_o_=10nm.** (***a***) MD simulation snapshots of 10% hydrogenated annulus, the gray domains represents boundaries which are not plotted in other figures for clarity. The inner and outer boundaries are fixed to keep still during relaxation and annulus with different H-coverage all have the same boundary radiuses. (***b***) The equilibrated wrinkled patterns around the inner rim after certain amount of rotation. (***c***) The failure mode of the hydrogenated annulus. (***d*-*f***) The wrinkle configurations of annulus with different hydrogen coverage *H*-coverage=0, 50%, 100% at rotation angle *∆θ*=10^o^.


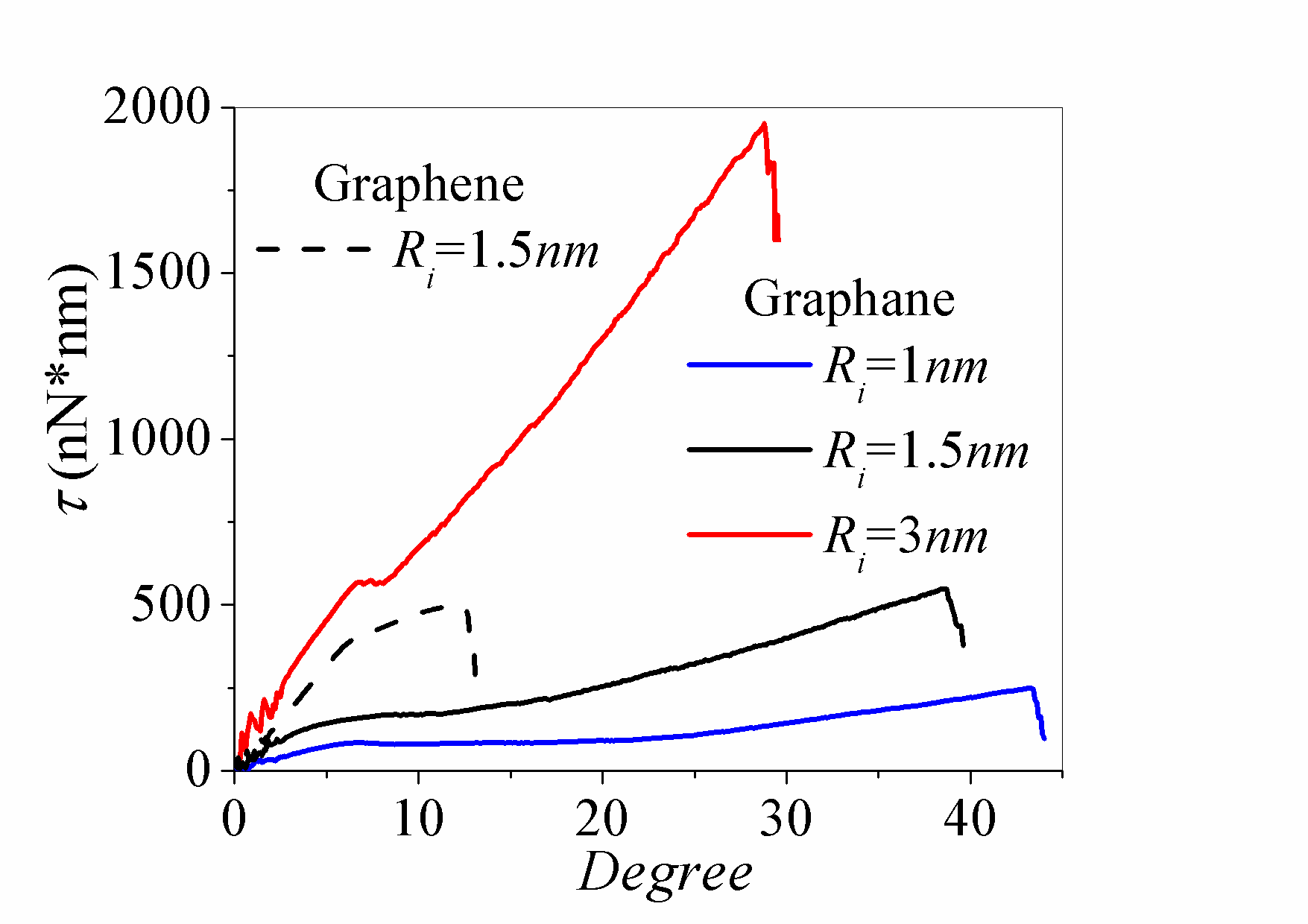


**Figure S5 | Evolution of the torque in annulus as a function of rotate angle Δ*θ* during the whole rotation process under fixed boundary condition.** The fluctuation of simulated results is improved compared to the results described in Figure 2.
