# Supplementary figures and images for: Surface hydrogenation regulated wrinkling and torque capability of hydrogenated graphene annulus under circular shearing
Source: Sci Rep. 2015 Nov 12;5:16556. doi: 10.1038/srep16556 (PMC4642312; doi:10.1038/srep16556)

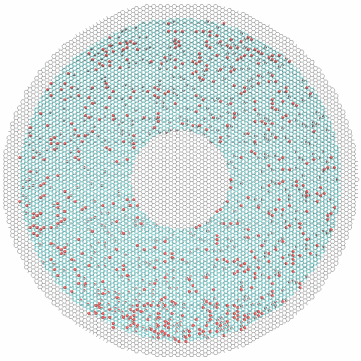

Supplement: Supplementary Video S1 [file srep16556-s2.gif]

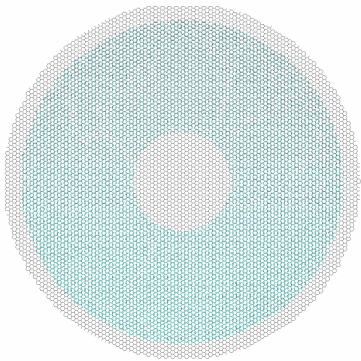

Supplement: Supplementary Video S2 [file srep16556-s3.gif]

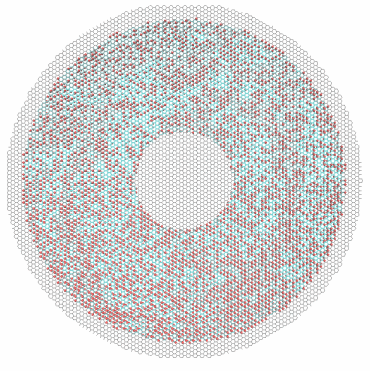

Supplement: Supplementary Video S3 [file srep16556-s4.gif]

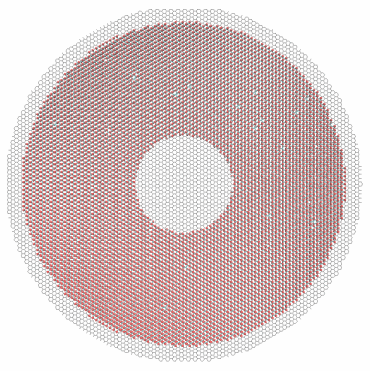

Supplement: Supplementary Video S4 [file srep16556-s5.gif]

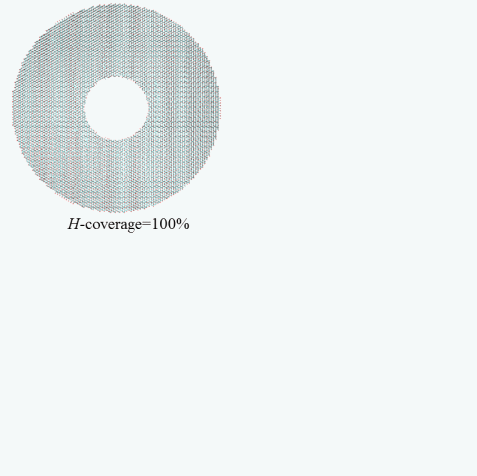

Supplement: Supplementary Video S5 [file srep16556-s6.gif]

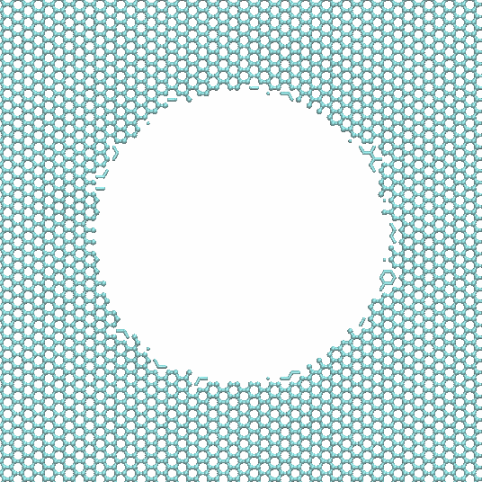

Supplement: Supplementary Video S6 [file srep16556-s7.gif]

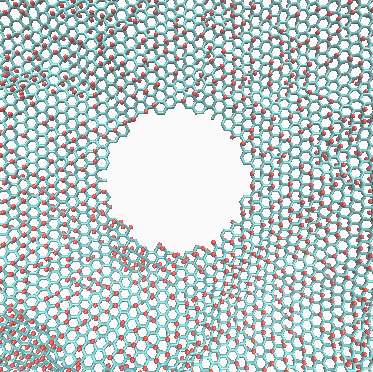

Supplement: Supplementary Video S7 [file srep16556-s8.gif]

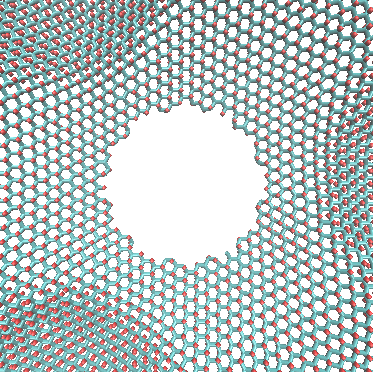

Supplement: Supplementary Video S8 [file srep16556-s9.gif]
